# Attractant and repellent induce opposing changes in the four-helix bundle ligand-binding domain of a bacterial chemoreceptor

**DOI:** 10.1371/journal.pbio.3002429

**Published:** 2023-12-11

**Authors:** Lu Guo, Yun-Hao Wang, Rui Cui, Zhou Huang, Yuan Hong, Jia-Wei Qian, Bin Ni, An-Ming Xu, Cheng-Ying Jiang, Igor B. Zhulin, Shuang-Jiang Liu, De-Feng Li

**Affiliations:** 1 State Key Laboratory of Microbial Resources, Institute of Microbiology, Chinese Academy of Sciences, Beijing, China; 2 University of Chinese Academy of Sciences, Beijing, China; 3 College of Resources and Environment, Huazhong Agricultural University, Wuhan, China; 4 National Laboratory of Biomacromolecules, CAS Center for Excellence in Biomacromolecules, Institute of Biophysics, Chinese Academy of Sciences, Beijing, China; 5 State Key Laboratory of Materials-Oriented Chemical Engineering, College of Biotechnology and Pharmaceutical Engineering, Nanjing Tech University, Nanjing, China; 6 Department of Microbiology, The Ohio State University, Columbus, Ohio, United States of America; Rutgers University-Robert Wood Johnson Medical School, UNITED STATES

## Abstract

Motile bacteria navigate toward favorable conditions and away from unfavorable environments using chemotaxis. Mechanisms of sensing attractants are well understood; however, molecular aspects of how bacteria sense repellents have not been established. Here, we identified malate as a repellent recognized by the MCP2201 chemoreceptor in a bacterium *Comamonas testosteroni* and showed that it binds to the same site as an attractant citrate. Binding determinants for a repellent and an attractant had only minor differences, and a single amino acid substitution in the binding site inverted the response to malate from a repellent to an attractant. We found that malate and citrate affect the oligomerization state of the ligand-binding domain in opposing way. We also observed opposing effects of repellent and attractant binding on the orientation of an alpha helix connecting the sensory domain to the transmembrane helix. We propose a model to illustrate how positive and negative signals might be generated.

## Abstract 2

How bacteria sense attractants is well understood, but how do they sense repellents? This study shows that although the attractant citrate and the repellent malate bind to the same site in the receptor ligand-binding domain of a Comamonas testosteroni chemoreceptor, they induce different conformational changes.

## Introduction

Bacteria utilize flagellar motility to navigate toward or away from spatial gradients of chemical stimuli [1]. This process, called chemotaxis, is vital for finding nutrients, escaping toxins, and establishing relationships with hosts [2,3]. Approximately half of known bacterial species have chemotaxis machinery genes encoded in their genomes [4], but the molecular mechanism of chemotaxis is best studied in the single model organism *Escherichia coli* where chemical signals are detected by ligand-binding domains (LBDs) of transmembrane chemoreceptors [5,6]. Chemoreceptor homodimers form mixed trimers-of-dimers [7–9], which are packed into a ternary hexagonal array with a histidine kinase CheA and a scaffolding protein CheW [10–12]. The decreased concentration of an attractant chemical detected by the chemoreceptor LBD promotes CheA autophosphorylation. Phosphorylated CheA donates its phosphoryl groups to the CheY response regulator, and the phosphorylated CheY (CheY-P) interacts with the flagellar motor and triggers the clockwise rotation resulting in tumbling [1]. Binding of an attractant to the chemoreceptor LBD suppresses CheA activity, which leads to dephosphorylation of CheY due to activity of its dedicated phosphatase CheZ and ultimately promotes swimming up the attractant gradient.

How small conformational changes resulting from an attractant binding propagate through the entire chemoreceptor molecule (several hundred Angströms) is not fully understood, although the role of structural and dynamic changes in various parts of the receptor was documented [13–17]. In *E*. *coli* Tar chemoreceptor, an attractant aspartate binds at the four-helix bundle LBD dimeric interface with a stoichiometry of one molecule per LBD homodimer [18] triggering an inward sliding of the last α-helix (α4) that extends into the second transmembrane helix (TM2) [5,19–21]. Consequently, transmembrane helices undertake piston and rotation movements and induce conformational changes in the HAMP domain that subsequently generate conformational changes in the downstream signaling domain altering CheA activity [22].

In contrast to the sensing mechanism for attractants, the molecular details of repellent recognition are poorly understood. In *E*. *coli*, Tar mediates a chemotactic repellent response to metal ions by an unknown mechanism [23], and no repellents that bind to Tar-LBD have been identified in large-scale screening [24]. *E*. *coli* chemoreceptor Tsr, which senses serine as an attractant, also senses another amino acid, leucine, as a repellent [25]. Most interestingly, a recent study showed that leucine and serine bind to the same binding pocket and a single amino acid substitution in the binding site converts the response to leucine from repellent to attractant [26]. Attractants and repellents cause the opposite behavior in chemotaxis. The in vivo FRET studies showed that addition of repellents increases CheA activity, whereas addition of attractants decreases the kinase activity [27,28], implying that attractants and repellents may also trigger the opposite conformational changes in chemoreceptors. Based on the observation that the attractant binding causes the α4 helix of Tar to move towards the cytoplasm by approximately 1.6 Å [20,21,29], it was proposed that repellent binding would cause an outward movement of one TM2 helix of the Tar dimer by 1 to 2 Å [13]. While an attractant causes the chemoreceptor dimers to move apart, away from each other [28], it was suggested that a repellent might cause the dimers to move closer to each other [30]. Nonetheless, these hypotheses have not been tested and how repellents trigger an opposite response from what attractants do remains unclear.

Previously, we reported that a transmembrane chemoreceptor MCP2201 in a gammaproteobacterium *Comamonas testosteroni* CNB-1 recognizes several tricarboxylic acid (TCA) cycle intermediates and mediates a positive chemotactic response towards these compounds. We also identified a binding site for an attractant citrate in the MCP2201 LBD [31], which, similarly to Tar-LBD and Tsr-LBD, adopts a four-helix bundle fold.

In this study, we identified malate as a repellent recognized by MCP2201. We show that malate binds to the same binding pocket that an attractant citrate (with only slight changes in interacting residues), but it affects LBD dimerization and the movement of the signaling α4 helix differently. Using chimeric proteins, we further show that the signal induced by malate binding to MCP2201 LBD can be transduced to cytoplasmic signaling domains of Tar and WspA chemoreceptors, causing negative chemotaxis to malate in *E*. *coli* and promoting biofilm formation in *Pseudomonas aeruginosa*, respectively.

## Results

### Malate is a repellent recognized by MCP2201 LBD

**Fig 1:**
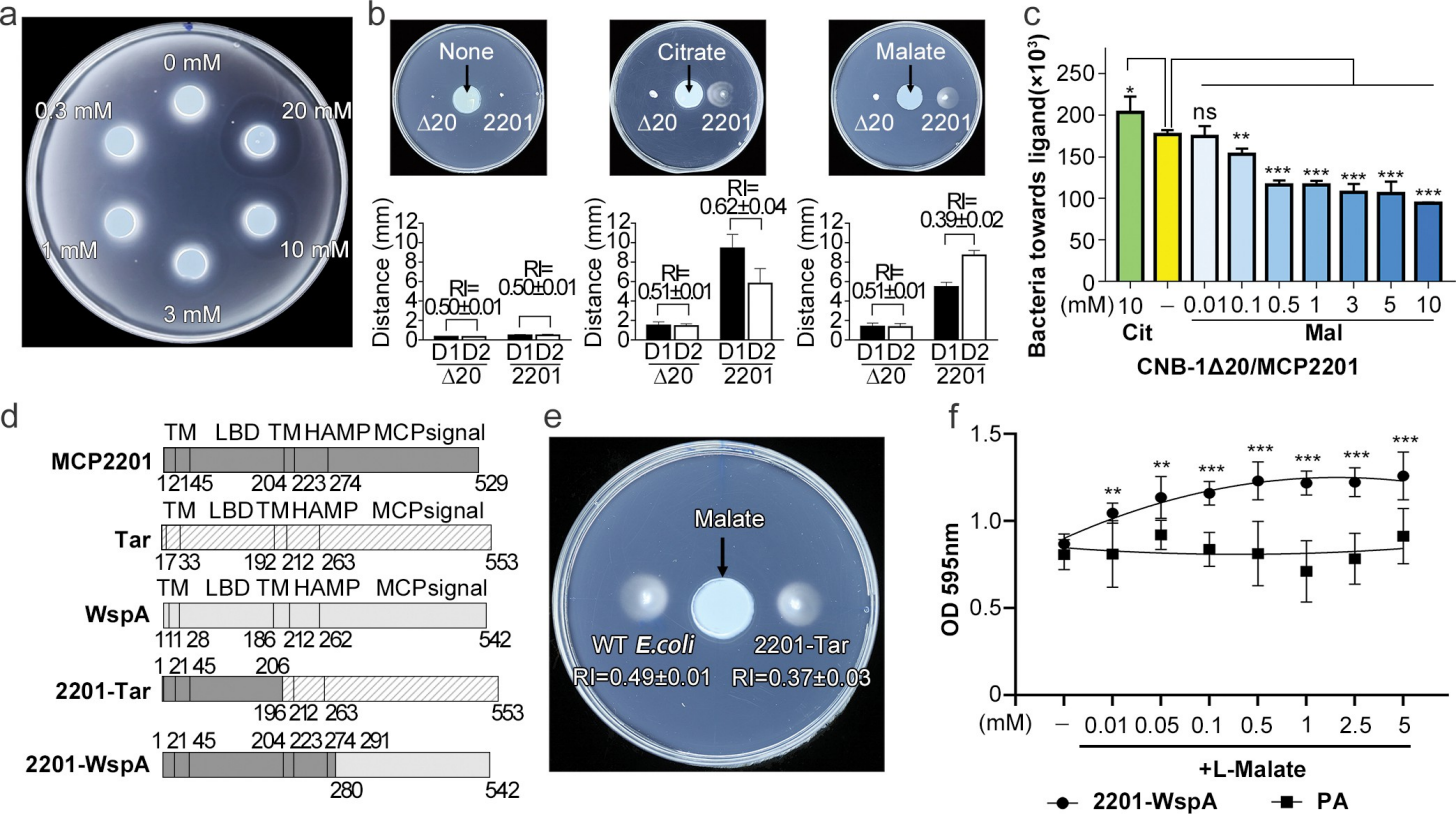
Chemotactic responses of CNB-1Δ20/MCP2201 and *E*. *coli* harboring MCP2201-Tar to malate and biofilm formation of *P*. *aeruginosa* harboring MCP2201-WspA. (**a**) Negative chemotaxis of CNB-1Δ20/MCP2201 cells in the presence of increasing concentrations of malate measured using the chemical-in-plug method. (**b**) Chemotactic responses of CNB-1Δ20/MCP2201 (labeled as 2201) and CNB-1Δ20 (labeled asΔ20) to citrate (10 mM) and malate (10 mM) in soft agar plate assay. No swarming was observed for cells with nothing in the plug (left panel), because there are no metabolizable attractants/repellents in the media. The RI was calculated as described in Materials and methods, and results are shown below each plate. Data are averages of 3 independent replicates. Error bars indicate standard deviations. The experiments in (**a**) and (**b**) were repeated for 3 times, and the representative examples were shown. (**c**) Chemotactic responses of CNB-1Δ20/MCP2201 to citrate and malate using the transwell chemotaxis assay. (**d**) Domain arrangement of MCP2201, Tar, WspA, and MCP2201–Tar and MCP2201-WspA chimeras. (**e**) Chemotaxis of *E*. *coli* strain harboring MCP2201-Tar (2201-Tar) or not (WT *E*. *coli*) to malate on the gradient soft-agar plate. *E*. *coli* did not grow on citrate; therefore, the assayed on citrate was not performed. (**f**) Biofilm formation of *P*. *aeruginosa* strains harboring MCP2201-WspA (2201-WspA) or not (PA) in the presence of varying concentrations of malate assessed using CV staining. Results are shown as the means ± SD (*n*   =  3). * (*P* < 0.05), ** (*P* < 0.01), and *** (*P* < 0.005) showed a significantly difference between ligand-treated and nontreated groups. The “ns” stands for not significant. The data underlying this Figure can be found in S1 Data. CV, crystal violet; HAMP, domain present in histidine kinases, adenylate cyclases, methyl-accepting chemotaxis proteins, and phosphatases; LBD, ligand-binding domain; MCP, methyl-accepting chemotaxis protein; PA, wild type Pseudomonas aeruginosa; RI, response index; TM, transmembrane; WT, wild type.

We used several approaches to demonstrate that malate is a repellent for *C*. *testosteroni*, which is recognized by chemoreceptor MCP2201. In the chemical-in-plug assay [23], the chemotaxis-null mutant CNB-1Δ20, in which all chemoreceptor genes have been deleted, complemented with a plasmid carrying wild-type MCP2201 gene (CNB-1Δ20/MCP2201), swam away from agar plugs containing 3, 10, or 20 mM L-malate, indicating that it is a repellent (Fig 1A). In the gradient plate assay [32], where chemotactic response index (RI) values greater than 0.52 indicate an attractant and those less than 0.48 indicate a repellent (see **Materials and methods** for details), CNB-1Δ20/MCP2201 strain responded to citrate with an RI value of 0.62 ± 0.04, indicating the attractant response (confirming the previous observation [33]), whereas an RI value for response to malate was 0.39 ± 0.02, indicating the repellent response (Fig 1B)). In the transwell chemotaxis assay, which is a modified version of a classic capillary method [34,35], more CNB-1Δ20/MCP2201 cells moved toward citrate compared to a buffer, and fewer cells moved towards increasing concentrations of malate, indicating that it acts as a repellent (Fig 1C).

Finally, we used chimera proteins to demonstrate that malate triggers a negative signal by MCP2201-LBD containing chemoreceptors. Functional chemoreceptor hybrids were successfully constructed in the past to demonstrate the common mechanism of transmembrane signaling in response to attractants by bacterial chemoreceptors and sensor histidine kinases [36]. We used a similar approach to construct chimeras consisting of the MCP2201 LBD and signaling domains from well-studied chemoreceptors in model organisms—Tar, which mediates an attractant response to aspartate in *E*. *coli* [37], and WspA chemoreceptor, which regulates biofilm formation in *P*. *aeruginosa* [38]. The MCP2201-Tar was constructed by fusing the MCP2201 region containing TM1, LBD, and partial TM2 and the Tar region containing partial TM2, HAMP domain, and the signaling (kinase control) domain. The MCP2201-WspA chimeras were constructed by fusing the MCP2201 region containing TM1, LBD, TM2, HAMP domain, and the signaling (kinase control) domain of WspA (Fig 1D). Differences in constructing these chimeras were dictated by the fact that the TM and HAMP domains of MCP2201 have lower similarity to Tar and higher similarity to WspA. *E*. *coli* MG1655 cells carrying the MCP2201-Tar chimera swam away from malate, with RI value of 0.37 ± 0.03 in a gradient soft-agar swim plate assay (Fig 1E), indicating that the transmembrane signal generated by MCP2201-LBD has been successfully transduced to the cytoplasmic domain of Tar. The MCP2201-WspA chimera was introduced into *P*. *aeruginosa* ΔWspA cells and the biofilm formation was evaluated by the crystal violet staining assay. Compared to the ΔWspA strain, cells complemented with MCP2201-WspA chimera produced more biofilm in the presence increasing concentrations of L-malate (Fig 1F), suggesting that MCP2201-WspA sensed malate and triggered the downstream biofilm formation signal. Taken together, results obtained by 4 independent methods demonstrate that malate is a repellent, which is sensed by MCP2201 chemoreceptor.

### Repellent malate binds to the same ligand binding pocket as does attractant citrate

**Fig 2:**
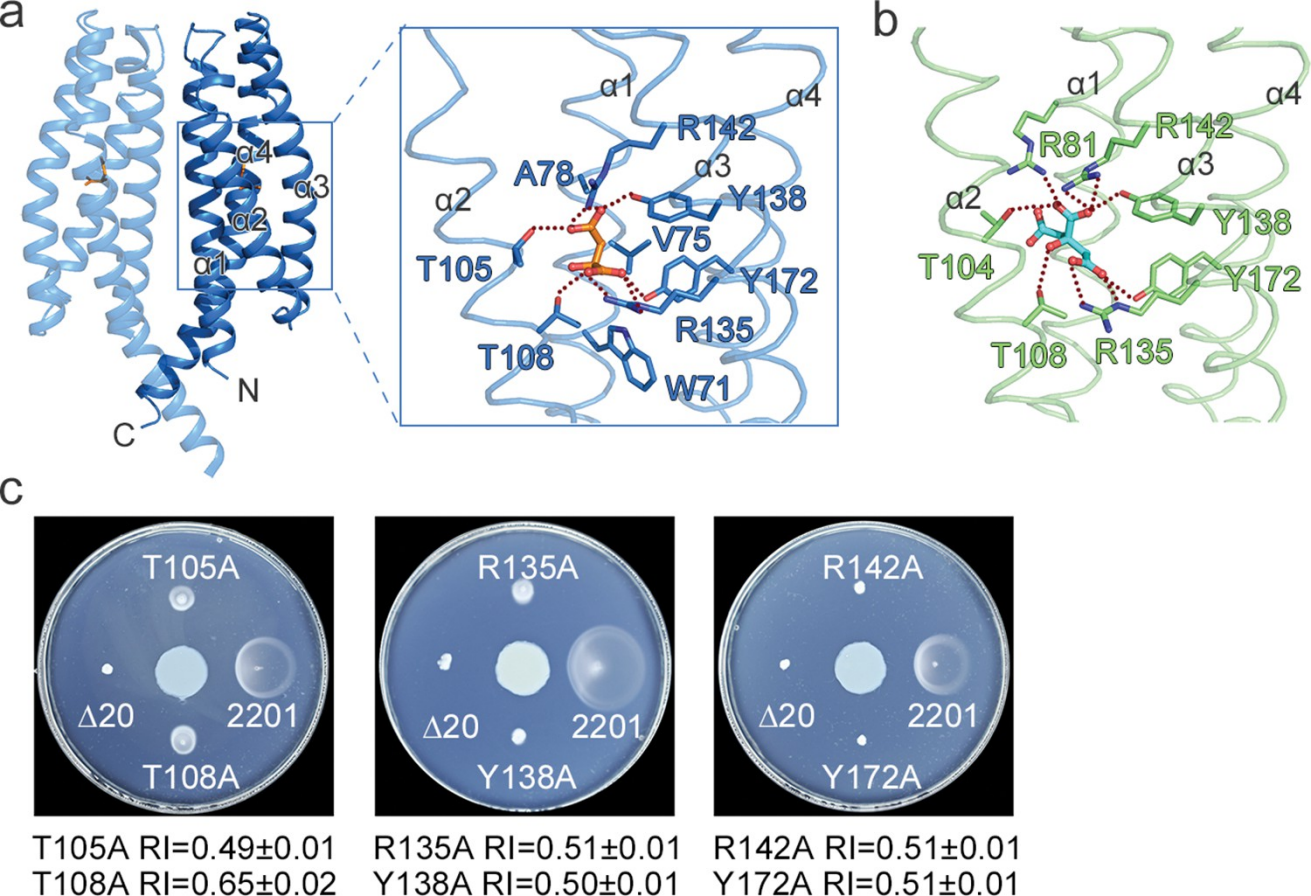
The MCP2201-LBD ligand-binding pocket and contact residues for malate and citrate binding. (**a**) Location of the binding pocket in MCP2201-LBD and residues involved in malate binding (brown stick representation, PDB accession code 7WR) and (**b**) residues involved in citrate binding (cyan stick representation, PDB accession code 6ITS). (**c**) Chemotactic responses to malate on the gradient soft-agar plates by CNB-1Δ20 cells harboring MCP2201 mutants. CNB-1 Δ20 harboring 2201 (2201) or not (Δ20) were used as controls. The experiments were repeated 3 times, and the representative examples are shown. The data underlying this Figure can be found in S1 Data.

We previously reported that MCP2201-LBD adopts a typical four-helix bundle fold and the attractant citrate binds at an internal pocket surrounded by all 4 helices [31]. Here, we determined the three-dimensional structure of MCP2201-LBD in complex with the repellent malate at 1.8 Å resolution and found a malate-bound MCP2201-LBD dimer in the asymmetric unit, similar to that observed in ligand-free MCP2201-LBD. As shown for the ligand-free and citrate-bound structures, the subunit of malate-bound MCP2201-LBD also folded into 4 helices (α1: Q59-K87; α2: A91-L118; α3: P122-A150; and α4: A154-E195). Malate was bound at an internal pocket surrounded by all 4 helices (Fig 2A), which overlaps with the citrate-binding pocket (Fig 2B). Specifically, residue R135 interacts with 1′-carboxyl group of malate via hydrogen bonds along with the potential interaction between negative and positive charges. Residue Y172 also interacts with the same carboxyl group. Residue T108 forms a hydrogen bond with the 2′-hydroxyl group of malate. Residues T105, Y138, and R142 form hydrogen bonds with the 4′-carboxyl group. Residues W71, V75, and A78 in helix α1 interact with malate via van der Waals interactions. The significant difference between malate- and citrate-binding pockets was that the ligand’s carboxyl group (coordinated by residues Y138 and R142 in both cases) forms a hydrogen bond with T105 in case of malate, but in case of citrate, this residue is not involved in ligand binding, but instead 2 other residues, R81 and T104, form hydrogen bonds with the ligand.

Residues involved in malate binding were subjected to mutagenesis, and the chemotaxis phenotypes of the resulted mutants were determined. Both the swimming plate assays reported in the previous study [31] and the current gradient plate assays showed that mutants Y138A, R142A, and Y172A lost the attractant response to citrate, whereas T108A and R135A did not (Fig A in S1 Text). The mutants T105A and R135A failed to respond to malate (RI 0.49 ± 0.01 and 0.51 ± 0.01, respectively), and phenotypes of Y138A, R142A, and Y172A were indicative of severe signaling defects (Fig 2C). Unexpectedly, the mutant T108A showed an attractant response to malate (moved towards malate, with RI of 0.65 ± 0.02; Fig 2C). We then verified malate binding affinities measured for the isolated periplasmic domains in T105A (no response) and T108A (inverted response) mutants and found that while the T105A mutant completely lost its ability to bind malate (the Kd was too weak to be determined), the T108A mutant bound malate with Kd of 995.1 ± 437.9 μM (Fig B in S1 Text). Thus, the T105A mutant showed significantly lower affinity for malate than the wild type MCP2201-LBD (Kd of 18.78 ± 7.45 μM), suggesting that the strength of ligand binding to chemoreceptors might play a critical role in chemotactic responses.

### Binding of attractant and repellent differently affects the LBD oligomeric state

**Fig 3:**
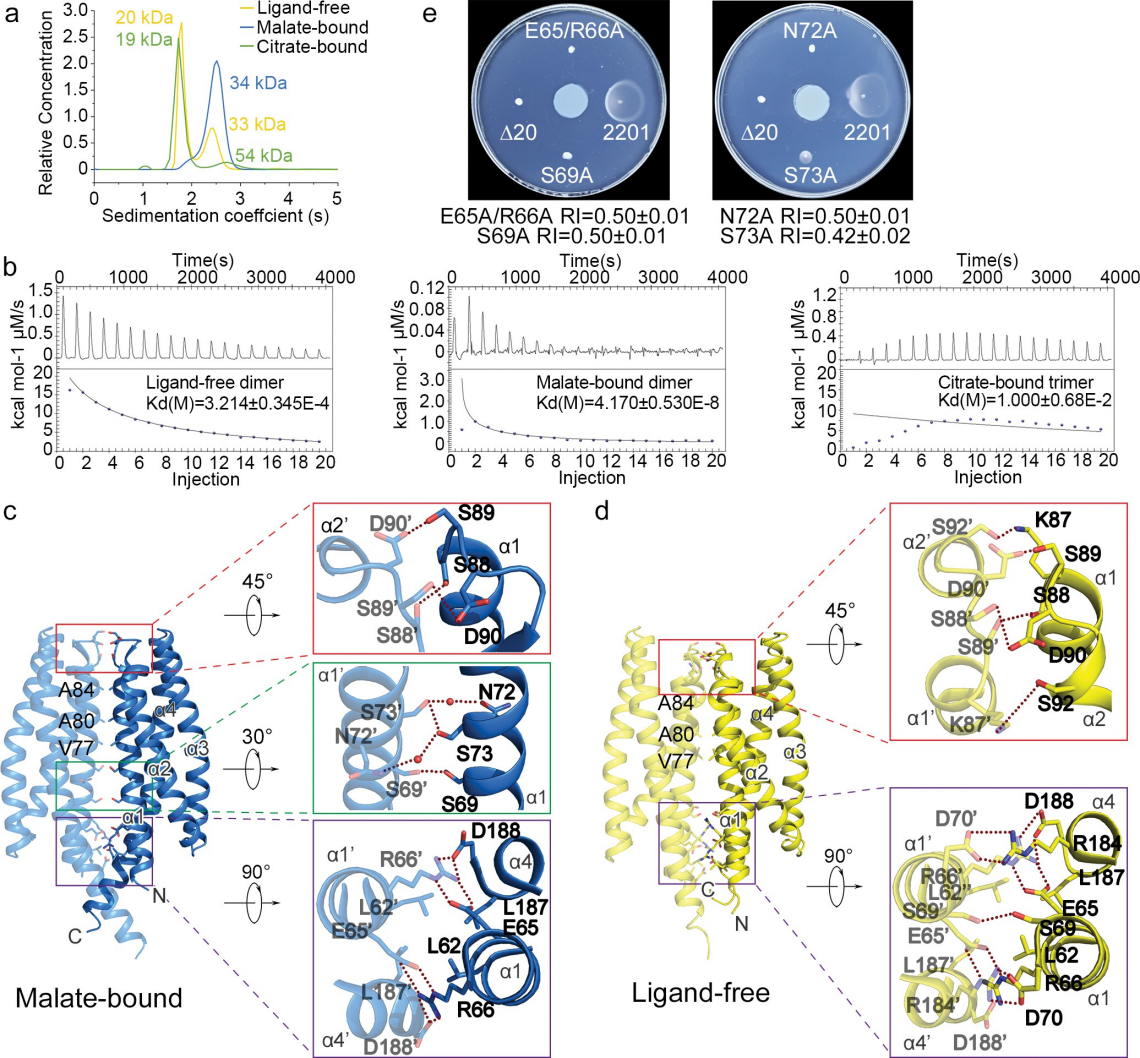
Oligomer states and dimeric interface of MCP2201-LBD. (**a**) Analytical ultracentrifugation assays of ligand-free, malate-bound (10 mM), and citrate-bound (10 mM) MCP2201-LBD. (**b**) ITC assays of MCP2201-LBD oligomer dissociation in the absence and in the presence of citrate and malate. Calorimetric dilution data (top) for injection of MCP2201-LBD (1.11 mM) without a ligand (left), in the presence of 10 mM malate (middle), and citrate (right) at 25°C were integrated, and dilution-corrected peaks were fitted to an oligomer dissociation model (bottom) to assess the dissociation constants. (**c**, **d**) The malate-bound (**c**, PDB accession code 7WRM) and ligand-free (**d**, PDB accession code 5XUA) dimers and residues involved in dimeric interface. Two subunits were colored in light and dark colors. (**e**) Chemotactic responses to malate on the gradient soft-agar plate by CNB-1Δ20 cells harboring MCP2201 mutants. The experiments in (**b**) and (**e**) were repeated for 3 times, and the representative examples were shown. The data underlying this Figure can be found in S1 Data.

Previous study revealed that the ligand-free and citrate-bound recombinant MCP2201-LBDs had different oligomeric states [31]. In this study, we investigated the oligomeric state of MCP2201-LBDs in the presence of repellent malate. Using analytical ultracentrifugation assays, we showed that, consistently with our previous observations [31], the citrate-bound MCP2201-LBDs primarily formed monomers (major fraction, approximately 90%, apparent molecular mass of 19 kDa), but also some trimers (minor fraction, approximately 10%, apparent molecular mass of 54 kDa) (Fig 3A), whereas the ligand-free MCP2201-LBDs was found in the equilibrium of monomer state (large fraction, apparent molecular mass of 20 kDa), and dimeric state (small dominant fraction, apparent molecular mass of 33 kDa) (Fig 3A). In contrast, the malate-bound MCP2201-LBDs primarily formed dimers (apparent molecular mass of 34 kDa) (Fig 3A). The oligomer disassociation constants of the ligand-free and malate-bound dimers were 32.14 ± 3.45 μM and 0.042 ± 0.005 μM, respectively, whereas the oligomer disassociation constant of citrate-bound trimers was hardly measurable, at approximately 10 mM (Fig 3B). The weak trimer affinity suggests the trimer may not be physiologically relevant (e.g., it could be caused by the truncation). The biological role of the trimer still remains uncertain and would be ignored in the following discussion since destabilizing the dimer interface is enough to propagate signals in the context of the full-length chemoreceptor dimer. The results suggest that binding of the repellent malate and the attractant citrate promote opposite changes in the MCP2201-LBD oligomerization state.

The crystal structures of the malate-bound and ligand-free MCP2201-LBD dimers were similar, with RMSD of 2.1 Å. The interface of malate-bound dimers was formed by helices α1 and α4 (Fig 3C and 3D). Residues L62, V77, A80, A84, and L187 from 2 subunits contributed to hydrophobic interactions and residues E65, R66, K87, S88, S89, D90, S92, R184, and D188 contributed to hydrogen bonds at the interface (Fig 3C). Residues S69, N72, and S73 were involved in a water-mediated hydrogen bond network at the malate-bound dimer interface, but not in the ligand-free dimer interface. The total buried area of malate-bound and ligand-free dimer was similar: approximately 1,280.7 Å^2^ and 1,281.1 Å^2^ per subunit, respectively. The free energies of malate-bound (−14.5 kcal/mol) and ligand-free (−10.8 kcal/mol) dimer formation calculated by PISA [39] support a more stable malate-bound dimer than the ligand-free one (Fig 3D).

In order to evaluate the importance of the dimeric interface, we first constructed 2 mutants, E65A/R66A and S73A, and showed that their malate-bound dimer disassociation constants were significantly higher (688.6 ± 409.5 μM for E65A/R66A and 954.8 ± 282.9 μM for S73A) (Fig B in S1 Text) than that of malate-bound wild-type MCP2201-LBD (0.042 ± 0.005 μM) (Fig 3B), indicating that the hydrogen bonds at the interface were critical for dimer formation. We then evaluated both mutants for their ability to respond chemotactically to malate and found that the chemotactic response was diminished in S73A mutant (RI value of 0.43 ± 0.02) and completely abolished in E65A/R66A mutant, which phenotype is suggestive of major signaling defect (Fig 3E), likely disrupting dimerization. Finally, we constructed 2 additional mutants, S69A and N72A, and both mutants showed no chemotactic response to malate, with phenotypes similar to that of E65A/R66A mutant (Fig 3E). Similar phenotypes were observed for mutants E65A/R66A, S69A, and N72A in response to citrate, suggesting a dimer disruption (Fig C in S1 Text). Notably, the S73A mutation did not alter the chemotactic respond to citrate, as shown in the previous study [31] and in this work. Taken together, these results suggest that the dimeric interface might be important for negative chemotaxis to malate mediated by MCP2201.

### Attractant and repellent induce a swing of the LBD signaling helix in opposite directions

**Fig 4:**
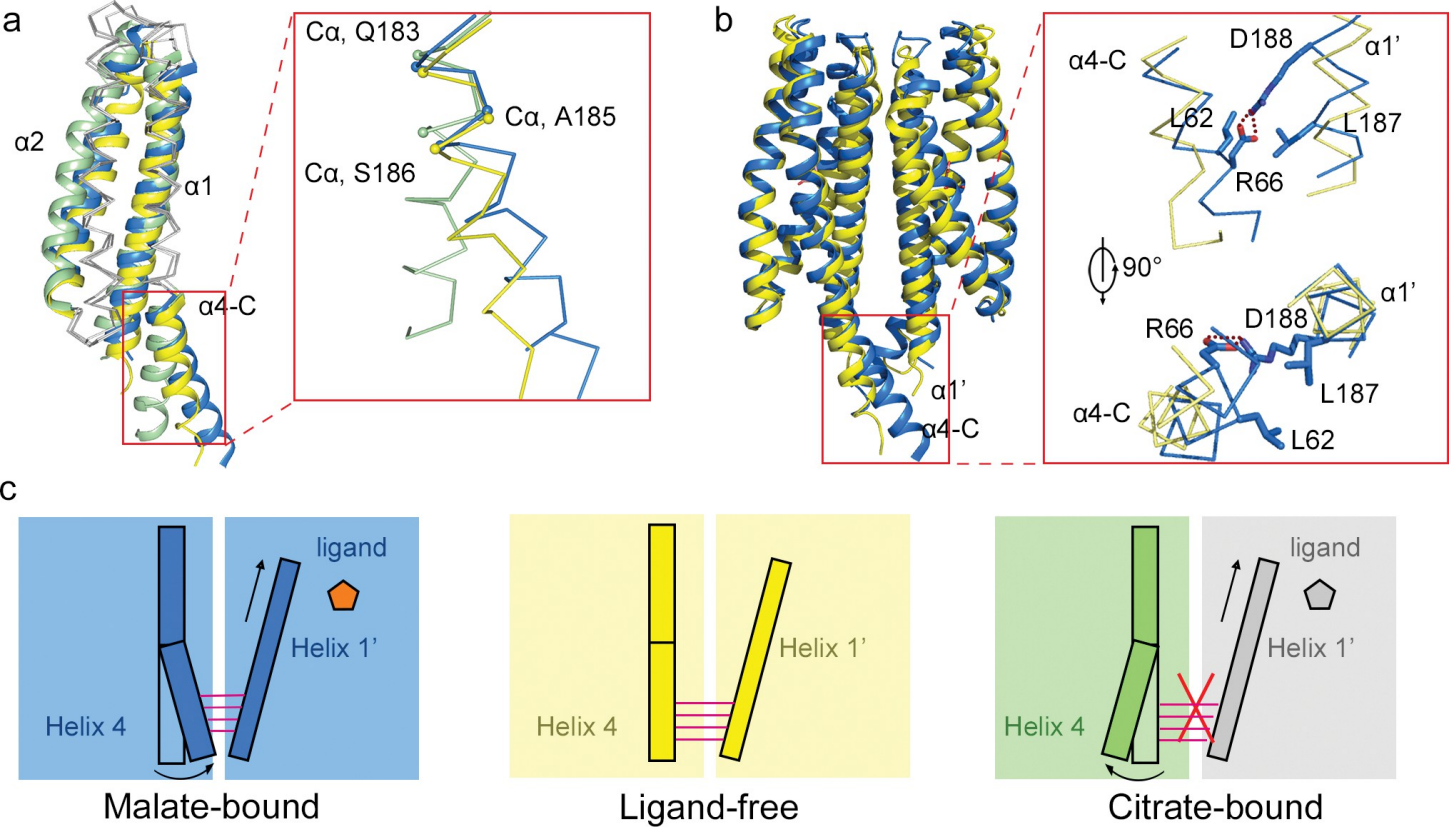
Position of C-terminus of helix α4 in ligand-free, malate-bound, and citrate-bound MCP2201-LBD. (**a**) Superposition of the ligand-free (yellow, PDB accession code 5XU), citrate-bound (green, PDB accession code 6IT), and malate-bound (blue, PDB accession code 7WRM) structures. The inset showed the different orientation of C-terminus of helix α4 in different structures. (**b**) Superposition of the ligand-free and malate-bound dimers. The inset showed the swing of C-terminus of helix α4 towards helix α1 of the other subunit and residues involved. (**c**) Cartoon representation of interaction between the C-terminus of helix α4 of one subunit and helix α1 of another subunit in ligand-free, malate-bound, and citrate-bound structures. Each color block represents a subunit. The upward arrow indicates a movement of helix α1, which is absent in the ligand-free state.

We superposed the ligand-free, citrate-bound, and malate-bound structures and noticed difference in positions of helices α1, α2, and the C-terminus of helix α4 (Fig 4A and 4B). Both malate and citrate interacted with residues W71, V75, and A78 in helix α1 via van de Waals interactions, inducing the displacement of helix α1 away from the membrane compared to the ligand-free dimer (Fig D in S1 Text). Malate formed hydrogen bonds with residues T105 and T108 in helix α2, whereas citrate formed hydrogen bonds with residues T104 and T108, resulting in different relative movement and bending angle variation of helices α2 (Fig D in S1 Text). Malate and citrate also formed hydrogen bonds with residues R135, Y138, R142, and Y172 in helices α3 and α4; however, we observed no significant conformational changes in helix α3 and in the N-terminal part of helix α4 (residues 154–182). In contrast, we observed a swing of the C-terminal part of helix α4 (residues 183–195) in ligand-bound structures compared to the ligand-free structure. The crystal structures of the 3 states (ligand-free, malate-bound, and citrate-bound) belong to different space groups and helix α4 did not participate in crystal stacking in any structure (Fig E in S1 Text). We therefore conclude that different conformations of helix α4 were caused by the ligands. Residue R66 in subunit one helix α1 formed hydrogen bonds with residue D188 in subunit two helix α4, and residue L62 in subunit one helix α1 formed hydrophobic interactions with residue L187 in subunit two helix α4. The movement of helix α1 induced a swing of the C-terminal part of helix α4 (residues 183–195). Furthermore, in malate-bound and citrate-bound structures, the swing was in opposing directions (Fig 4A). The C-terminal part of helix α4 is the truncation point for this construct; thus, it is important to emphasize that conformational changes upon ligand binding might be different in the context of the intact chemoreceptor. We have to mention that the lattice contacts on other surfaces might influence the conformation of helix α4. The ligand-free, malate-bound, and citrate-bound structures belonged to different space groups, which might influence helix α4 via different crystal packings. Thus, we cannot completely rule out the possibility of that the structures were altered by the crystal packing in different space groups.

## Discussion

Molecular mechanisms of detecting chemical attractants by bacterial chemoreceptors have been studied extensively [19,22,29,40]. In a striking contrast, the detection mechanisms for repellents remain poorly understood. In this study, we identify malate as a repellent recognized by MCP2201 chemoreceptor of *C*. *testosteroni* and demonstrate that an attractant and a repellent induce opposing changes in its four-helix bundle LBD.

We propose the following model for observed opposing conformational changes in MCP2201-LBD upon attractant and repellent binding. In the ligand-free structure, residues 183–195 in helix α4 of one subunit are packed against residues 57–70 in helix α1 of the other subunit via hydrogen bonds and van de Waals interactions (Fig D in S1 Text), thus constraining their conformations and contributing to the dimeric interface. Malate binding strengthens the dimer organization. Helix α1 moves away from the membrane and then induces the swing of residues 183–195 in helix α4 of the other subunit towards the direction where helix α1 moves via interdimer interaction (Fig 4B). In contrast, upon citrate binding, the movement of helix α1 causes a steric hindrance that restricts dimer formation (Fig F in S1 Text) [31]. The citrate-bound MCP2201-LBD exists dominantly as the monomer [31]. In the monomeric state, residues 181–195 are not involved in any interface packing, thus contributing to the different orientation of the C-terminal part of helix α4 (Fig 4C).

Several studies reported that the attractant binding alters the oligomeric state of chemoreceptor LBDs, including the 4HB_MCP domains of *E*. *coli* Tar [18,41–43], *Pseudomonas putida* PcaY_ [44] and *P*. *aeruginosa* CtpH [45], the HBM domains of *P*. *putida* McpS [46] and McpQ [47], and the PilJ domain of *P*. *aeruginosa* McpN [48], all of which adopt the four-helix bundle fold. It was suggested that ligand binding at the dimeric interface alters the oligomeric state because ligands interact with both subunits. On the other hand, no such alteration was seen in Cache domains [49] where attractants bind to a defined pocket in each subunit. For example, in *P*. *aeruginosa*, the dCache_1 domain of PctA is found as a monomer in both ligand-free and ligand-bound states [50], and the sCache_2 domain of PA2652 exists as a dimer in both states [51]. MCP2201-LBD represents the third kind of chemoreceptor binding domains, where ligands do not bind at the dimeric interface of the four-helix bundle LBD but do alter its oligomerization. We found the attractant binding shifts the ligand-free weak MCP2201-LBD dimer state (an equilibrium of monomer and homodimer states) to the dominant monomer state, whereas the repellent binding shifts it to the stable dimer state. These results were obtained using isolated recombinant LBDs, and we do not know how the observed changes would propagate through the TM2, the HAMP domain, and the signaling domain. Signaling domains of diverse chemoreceptors form stable trimers of dimers [52], and no evidence for changes in LBD oligomeric state come from in-cell tomography studies. Undoubtedly, stable trimers of dimers put severe constraint on the LBD movement, and free transitions between various oligomeric states reported here are highly unlikely to occur in full-length chemoreceptors. However, our results suggest that opposing conformational changes in LBD induced by an attractant and a repellent will translate in opposing downstream signal propagations.

The major structural difference between ligand-free, citrate-bound, and malate-bound MCP2201-LBD include a piston movement of helix α1, a bending of helix α2, and a swing movement of C-terminus of helix α4. We propose that the malate binding induces the displacement of helices α1, modifies the dimeric interface, increases the dimer formation, and then alters the orientation of the helix α4 C-terminus (via the dimeric interface interaction between helix α1 in one subunit and α4 in the other subunit). In contrast, the citrate binding disrupts the dimeric interface, thereby increasing the conformational freedom of residues involved in ligand-free and malate-bound dimer formation and altering the orientation of the C-terminus of helix α4. The proposed mechanism is different from the canonical *E*. *coli* model, and it will require substantial work to support or refute this hypothesis. However, whether the signal comes from one monomer or another, the downstream signaling, which involves TM2 and the HAMP domain, would likely be the same. Our results with Tar and WspA support (albeit indirectly) this suggestion.

Differences in ligand-induced conformational changes reported in *E*. *coli* Tar and Tsr LBDs and observed here in MCP2201-LBD are not necessarily surprising. All 3 domains belong to the same superfamily, which the leading protein domain database InterPro [53] classifies as “Four helix bundle sensory module for signal transduction” (InterPro accession cl0457). However, within this superfamily, MCP2201 LBD belongs to the largest family that represents approximately 50K of proteins and approximately 14K of bacterial and archaeal species (InterPro accession IPR024478), whereas Tar and Tsr LBDs are found in a smaller family of approximately 15K of proteins and approximately 4K of species (InterPro accession IPR003122). InterPro protein families are defined by sequence similarity, thus MCP2201-LBD and Tar/Tsr LBDs are only distantly related despite sharing the same fold.

Despite remote homology, Tsr and MCP2201 share a remarkable common property. Using molecular docking and mutation experiments, a recent study revealed that the repellent leucine binds to the same binding site on Tsr-LBD as the attractant serine, and only minor changes in 1 or 2 amino acid residues in the LBD determine whether the ligand induces attractant or repellent response [26]. The single amino acid substitution in the binding pocket reversed the response to leucine from negative to positive chemotaxis [26]. Here, we reveal a similar case, where intermediates of the TCA cycle citrate and malate bind to the same binding site of MCP2201-LBD, causing an attractant and a repellent response, respectively. Furthermore, as in the case of Tsr response to leucine, a single amino acid substitution in the binding pocket of MCP2201-LBD converted the response to malate from negative to positive chemotaxis. This apparent similarity is striking, because the location of binding sites is quite different: Serine and leucine bind at the dimer interface of Tsr-LBD, whereas citrate and malate bind inside the MCP2201-LBD monomer.

Over a hundred of different LBD types were identified in bacterial chemoreceptors [54], but all chemoreceptors contain only one type of a signaling domain [55], and membrane topology Class I, where extracytoplasmic LBD is flanked by 2 TM helices, is predominant in chemoreceptors [56] and widespread in sensor histidine kinases. Consequently, signal transduction from extracytoplasmic LBDs to signaling domains involves their shared structural elements—a helix adjacent to TM2, TM2 itself, and the downstream HAMP domain [13]. Conformational changes leading to signaling by LBDs of various types are likely to be different. How they are translated into a universal signal modulating CheA kinase remains to be explored.

## Materials and methods

### Bacterial strains, plasmids, media, and growth conditions

Bacterial strains and plasmids used in this study are listed in Table A in S1 Text. Genetic complementation in *C*. *testosteroni* CNB-1 and *E*. *coli* MG1655 was conducted using pBBR1MCS-2 and that in *P*. *aeruginosa* ΔWspA using pHERD20T. *C*. *testosteroni* CNB-1 was cultivated in LB broth at 30°C, and *E*. *coli* MG1655 and *P*. *aeruginosa* ΔWspA strains in LB broth at 37°C.

### Site-directed mutagenesis and chimera construction

The DpnI-mediated site-directed mutagenesis was conducted as previously described [31]. The MCP2201-Tar and MCP2201-WspA chimeras were constructed using overlapping PCR method, similar to that used for NarX-Tar chimera [36].

### Chemical-in-plug assay

Gradient soft-agar swim plate assays were performed as previously described with minor changes [23]. Briefly, chemoeffector-containing agar plugs were prepared by mixing molten 1.5% (wt/vol) agar with 0, 0.3, 1, 3, 10, and 20 mM malate and placed into the Petri dish. *C*. *testosteroni* CNB-1 was grown in LB medium to OD_600_ of 0.5 to 0.7, washed, and resuspended in Chemotaxis buffer (40 mM NaH2PO4, 10 μM EDTA, 0.05% glycerol (pH 7.5)) and MSB medium (1 g/L Na_2_HPO_4_·12H_2_O, 0.5 g/L KH_2_PO_4_, 0.03 g/L MgSO_4_·7H_2_O, and 1 g/L NH_4_CL), respectively. Bacterial suspensions were poured into Petri dishes and incubated at room temperature for 2 to 10 min and then examined to identify the clearing zone around the plug.

### Gradient soft-agar swim plate assay

Semisolid-agar assays were performed as previously described [32,57]. Briefly, chemoeffector-containing agar plugs were prepared by mixing molten 1.5% (wt/vol) agar with 10 mM L-malate or citrate and placed into the center of the Petri dish containing the semisolid-agar with 0.25% (wt/vol) agarose in MSB medium (1 g/L Na_2_HPO_4_·12H_2_O, 0.5 g/L KH_2_PO_4_, 0.03 g/L MgSO_4_·7H_2_O, and 1 g/L NH_4_CL). *C*. *testosteroni* CNB-1 cells were grown in LB medium to OD_600_ of 0.8, washed, and resuspended in 50 μl MSB medium. Around 0.5 μl of bacterial suspensions were inoculated 2 cm away from the center of the ligand plug. The assay plates were incubated at 30°C for 24 h. For *E*. *coli* MG1655 cultures, the media for plate assay contained 10 mM KPO_4_ (pH 7.0), 1 mM (NH_4_)_2_SO_4_, 1 mM MgCl_2_, 1 mg/L thiamine HCl, 0.1 mM threonine, methionine, leucine, and histidine, and cells were inoculated 2.5 cm away from the center of the plug and incubated at 37°C for 24 h. The distances from the inoculation sites to the colony edges closest to (D1) and furthest from (D2) the agar plug center were measured and the RI values were calculated as follows: RI = D1/(D1 + D2), as described previously [32]. Attractant and repellent were defined when RI values greater than 0.52 and less than 0.48, respectively.

### Transwell chemotaxis assay

Transwell chemotaxis assay were performed as previously described [34,35]. The experimental setup consists of a cylindrical top well insert with transparent PET membrane placed in 24-well plate. Briefly, *C*. *testosteroni* CNB-1 cells were grown in LB medium to OD_600_ of 0.5 to 0.7, washed, and resuspended in chemotaxis buffer (40 mM NaH2PO4, 10 μM EDTA, 0.05% glycerol (pH 7.5)) or MSB medium (1 g/L Na2HPO4·12H2O, 0.5 g/L KH2PO4, 0.03 g/L MgSO4·7H2O, and 1 g/L NH4CL), respectively. Around 700 μl of MSB medium containing different concentrations of citrate or malate were added to 24-well cell culture plates. Add 300 μl of the bacterial suspensions into the top well. After incubation at 30°C for 60 min, the number of cells that entered each well was calculated (in CFUs/mL) by considering the dilution factor.

### Protein expression and purification

The coding sequence for MCP2201-LBD (residues 58–203) was cloned in pET22b expression vector (Novagen) with an N-terminal His_6_-tag. The constructed plasmids were transformed into *E*. *coli* BL21 (DE3). The cells were cultured in LB medium with 50 μg/ml kanamycin at 37°C to an OD_600_ of 0.8 to 1.0. Protein expression was induced with 0.2 mM IPTG at 25°C for 10 h. Bacterial cells were harvested by centrifugation at 5,000*g* for 30 min. The cell pellet was resuspended in lysis buffer consisting of 50 mM Tris buffer (pH 7.5), 200 mM NaCl, and 10 mM imidazole. The cells were lysed on ice by sonication and then centrifuged at 20,000*g* for 30 min. The supernatant was incubated with nickel affinity resins (Ni-NTA, Qiagen) at 4°C for 30 min. These resins were washed 3 times with washing buffer containing 20 mM Tris (pH 7.5), 1 M NaCl, and 20 mM imidazole. Protein was eluted with elution buffer containing 20 mM Tris (pH 7.5), 200 mM NaCl, and 250 mM imidazole. The eluted protein was loaded on a HiTrap Q HP column (GE Healthcare) in buffer consisting of 20 mM Tris (pH 7.5) and 150 mM (SEC buffer) and eluted using SEC buffer supplied with 1 M NaCl. The elution was then loaded on a Superdex 200 10/300 GL column (GE Healthcare) and eluted in the SEC buffer. The fractions containing pure protein were concentrated to 10 mg/ml for crystallization screening in SEC buffer.

### Crystallization, data collection, and structure determination

L-malate-bound MCP2201LBD crystals were obtained using the hanging-drop vapor diffusion method at 289 K, by mixing equal volumes of protein (10 mg/ml) and reservoir solution that contained 0.2 M ammonium acetate, 0.1 M Bis–Tris (pH 5.6), 22% PEG 3,350, 5% glycerol. The best crystals were transferred to mother liquor containing 20% glycerol as a cryoprotectant and then flash frozen in liquid nitrogen. The diffraction datasets were collected at beamline BL19U1, Shanghai Synchrotron Radiation Facility (SSRF, China). The data were processed and scaled with the iMOSFLM [58], XDS [58], and/or CCP4 suite [59]. The structure was solved by molecular replacement with Phaser in the PHENIX suite [60], and the structure of ligand-free MCP2201LBD (PDB entry 5XUA) was employed as a search model. The L-malate-bound MCP2201LBD structure was built by PHENIX AutoBuild and refined with PHENIX and Coot [61]. The data collection and refinement statistics are summarized in Table B in S1 Text. Figures were prepared with PyMOL (http://www.pymol.org).

### Analytical ultracentrifugation assay

MCP2201-LBD was diluted to 75 μM in SEC buffer with or without 10 mM ligand. Sedimentation velocity analytical ultracentrifugation experiments were performed on a Beckman XL-I analytical ultracentrifuge (Beckman Coulter, Brea, California, United States of America) at 4°C with a rotor speed of 60,000 rpm for 7 h. Results were analyzed using Sednterp, and the sedimentation coefficients and apparent molecular masses were calculated as previously described [62].

### Isothermal titration calorimetry

ITC experiments were performed at 25°C using Affinity ITC (TA Instruments). For dimer dissociation assays, 1.1 mM proteins dissolved in SEC buffer with or without 10 mM ligand were injected into the sample cell containing the identical buffer mixture. For ligand-binding assays, 100 μM proteins were introduced into the sample cell and titrated with ligand dissolved in SEC buffer. Data were analyzed with the NanoAnalyze software package using independent model (TA Instruments, New Castle, USA).

### Biofilm formation assay

Biofilm formation assay was performed following a previously published protocol with minor modifications [63]. Strain *P*. *aeruginosa* ΔWspA mutant complemented with MCP2201-WspA chimera strain was grown overnight in LB, washed, and diluted to the OD_600_ of 0.008 in Jensen medium. The effect of L-malate on biofilm formation was examined by incubation with varying concentrations of L-malate (0, 10 μM, 50 μM, 100 μM, 500 μM, 1 mM, 2.5 mM, 5 mM) in the diluted cells. Approximately 100 μl aliquots of the diluted cells were pipetted into wells of a sterile 96-well U-bottom microtiter plate. After incubation at 30°C for 24 h without shaking, attached cells were washed with ddH_2_O and then stained with 0.1% crystal violet solution. The crystal violet was then dissolved in 200 μl of 40% acetic acid, and its absorbance at 595 nm was measured. Experiments were repeated for 3 times.

## Supporting information

S1 Text. Fig A Chemotactic responses to citrate on the gradient soft-agar plate by CNB-1Δ20 cells harboring MCP2201 mutants. CNB-1 Δ20 harboring 2201 (2201) or not (Δ20) were used as controls. The experiments were repeated for 3 times, and the representative examples were shown. The data underlying this Figure can be found in S1 Data. **Fig B. Mutations in the ligand-binding pocket affect L-malate binding.** Typical raw titration curves of MCP2201LBD (wild type), T105A and T108A mutant (0.1 mM) for L-malate (1 mM) binding at 25°C. Integrated and normalized heat signals versus molar ratio are listed at the bottom. The data underlying this Figure can be found in S1 Data. **Fig C. Mutations in the dimeric interface affect dimer dissociation.** Calorimetric dilution data for the dissociation of E65A/R66A, S69A, and A84W mutant MCP2201LBD dimers in the presence of L-malate. Raw titration data for injection of MCP2201LBD (1.11 mM) into 10 mM L-malate solution at 25°C are shown at the top; integrated and dilution corrected peaks are fit to a dimer dissociation model and are shown at the bottom. The data underlying this Figure can be found in S1 Data. **Fig D. Structural comparison of apo-, citrate-bound, and malate-bound MCP2201-LBD.** (**a**) Movements of residues T104, T105, and T108 of helix α2 towards the ligand and the bend angles of the C-terminus of helix α2 in apo- (yellow, PDB accession code 5XUA), malate-bound (blue, PDB accession code 7WRM), and citrate-bound (green, PDB accession code 6ITS) structures. (**b**) Displacements of helix α1 away from the membrane upon ligand binding. (**c**) Packing of the N-terminus of helix α1 and the C-terminus of helix α4 in the dimeric interface. **Fig E. The crystal packing of the 3 LBD states**. Ligand-free (PDB entry 5XUA), malate-bound (PDB accession code 7WRM), and citrate-bound MCP2201LBD (PDB accession code 6ITS) were shown. Blue box represents the crystal cell, each color represents the asymmetric unit, and the red color represents the C-terminus of helix α4. **Fig F. Citrate binding induces steric hindrance preventing MCP2201 dimerization.** Two citrate-bound MCP2201LBD monomers (green, PDB accession code 6ITS) are superposed with an L-Malate-bound dimer (blue, PDB accession code 7WRM). Two steric hindrances are identified by red circles: Val77 and the loop between helix α1 and α2. **Table A. Strains and plasmids used in this study. Table B. Data collection and refinement statistics for MCP2201LBD structure.**
(PDF)

S1 Data The numerical values underlying those figures in main text and supporting information are deposited in S1_Data.xlsx file. The data include the following: (1) chemotactic responses of CNB-1Δ20/MCP2201 and CNB-1Δ20 in Fig 1B; (2) chemotactic responses of CNB-1Δ20/MCP2201 to citrate and malate using the transwell chemotaxis assay in Fig 1C; (3) chemotaxis of *E*. *coli* strain harboring MCP2201-Tar (2201-Tar) or not (WT *E*. *coli*) to malate on the gradient soft-agar plate in Fig 1E; (4) biofilm formation of *P*. *aeruginosa* strains harboring MCP2201-WspA (2201-WspA) or not (PA) in Fig 1F; (5) chemotactic responses to malate on the gradient soft-agar plates by CNB-1Δ20 cells harboring MCP2201 mutants in Fig 2C; (6) analytical ultracentrifugation assays of ligand-free, malate-bound (10 mM), and citrate-bound (10 mM) MCP2201-LBD in Fig 3A; (7) ITC assays of MCP2201-LBD oligomer dissociation in the absence and in the presence of citrate and malate in Fig 3B; (8) chemotactic responses to malate on the gradient soft-agar plate by CNB-1Δ20 cells harboring MCP2201 mutants in Fig 3E; (9) ITC assays of MCP2201-LBD mutants’ malate affinity in Fig B; (10) ITC assays of MCP2201-LBD mutants’ oligomer dissociation in the presence of malate in Fig C; (11) chemotactic responses to citrate on the gradient soft-agar plate by CNB-1Δ20 cells harboring MCP2201 mutants in Fig A.
(XLSX)

## Abbreviations

CV: crystal violet
HAMP: domain present in histidine kinases, adenylate cyclases, methyl-accepting chemotaxis proteins, and phosphatases
LBD: ligand-binding domain
MCP: methyl-accepting chemotaxis protein
PA: wild type Pseudomonas aeruginosa
RI: response index
TCA: tricarboxylic acid
TM: transmembrane
WT: wild type
