# Supplementary material for: Attractant and repellent induce opposing changes in the four-helix bundle ligand-binding domain of a bacterial chemoreceptor
Source: PLoS Biol. 2023 Dec 11;21(12):e3002429. doi: 10.1371/journal.pbio.3002429 (PMC10735184; doi:10.1371/journal.pbio.3002429)
Supplement: S1 Text. Fig A — CNB-1 Δ20 harboring 2201 (2201) or not (Δ20) were used as controls. The experiments were repeated for 3 times, and the representative examples were shown. The data underlying this Figure can be found in S1 Data. Fig B. Mutations in the ligand-binding pocket affect L-malate binding. Typical raw titration curves of MCP2201LBD (wild type), T105A and T108A mutant (0.1 mM) for L-malate (1 mM) binding at 25°C. Integrated and normalized heat signals versus molar ratio are listed at the bottom. The data underlying this Figure can be found in S1 Data. Fig C. Mutations in the dimeric interface affect dimer dissociation. Calorimetric dilution data for the dissociation of E65A/R66A, S69A, and A84W mutant MCP2201LBD dimers in the presence of L-malate. Raw titration data for injection of MCP2201LBD (1.11 mM) into 10 mM L-malate solution at 25°C are shown at the top; integrated and dilution corrected peaks are fit to a dimer dissociation model and are shown at the bottom. The data underlying this Figure can be found in S1 Data. Fig D. Structural comparison of apo-, citrate-bound, and malate-bound MCP2201-LBD. (a) Movements of residues T104, T105, and T108 of helix α2 towards the ligand and the bend angles of the C-terminus of helix α2 in apo- (yellow, PDB accession code 5XUA), malate-bound (blue, PDB accession code 7WRM), and citrate-bound (green, PDB accession code 6ITS) structures. (b) Displacements of helix α1 away from the membrane upon ligand binding. (c) Packing of the N-terminus of helix α1 and the C-terminus of helix α4 in the dimeric interface. Fig E. The crystal packing of the 3 LBD states. Ligand-free (PDB entry 5XUA), malate-bound (PDB accession code 7WRM), and citrate-bound MCP2201LBD (PDB accession code 6ITS) were shown. Blue box represents the crystal cell, each color represents the asymmetric unit, and the red color represents the C-terminus of helix α4. Fig F. Citrate binding induces steric hindrance preventing MCP2201 dimerization. Two citrate-bound MCP2201LBD mo [file pbio.3002429.s001.pdf]

Supporting Information

Attractant and repellent induce opposing changes in the four-helix bundle ligand-binding domain of a bacterial chemoreceptor

Lu Guo[1,2#], Yun-Hao Wang[1,2,3#], Rui Cui[1,2#], Zhou Huang[1], Yuan Hong[4], Jia-Wei Qian[1], Bin Ni[1], An-Ming Xu[5], Cheng-Ying Jiang[1], Igor B. Zhulin[6]*, Shuang-Jiang Liu[1,2]*, De-Feng Li[1,2]*

Contents

Figures A to F

Tables A to B

Legend for Data S1

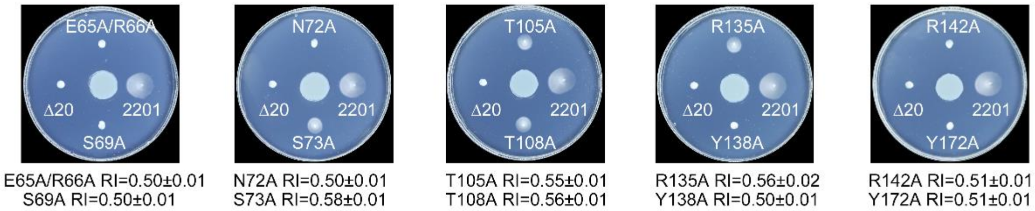

Fig A. Chemotactic responses to citrate on the gradient soft-agar plate by CNB-1Δ20 cells harboring MCP2201 mutants. CNB-1 Δ20 harboring 2201 (2201) or not (Δ20) were used as controls. The experiments were repeated for three times, and the representative examples were shown. The data underlying this Figure can be found in S1 Data.

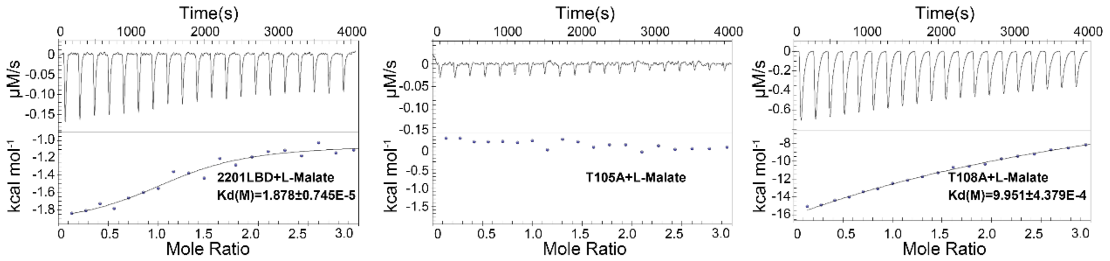

Fig B. Mutations in the ligand-binding pocket affect L-malate binding. Typical raw titration curves of MCP2201LBD (wild type), T105A and T108A mutant (0.1 mM) for L-malate (1 mM) binding at 25 °C. Integrated and normalized heat signals versus molar ratio are listed at the bottom. The data underlying this Figure can be found in S1 Data.

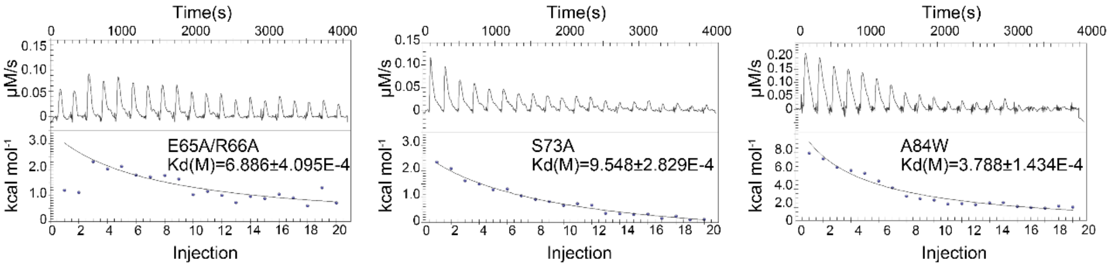

Fig C. Mutations in the dimeric interface affect dimer dissociation. Calorimetric dilution data for the dissociation of E65A/R66A, S69A, and A84W mutant MCP2201LBD dimers in the presence of L-malate. Raw titration data for injection of MCP2201LBD (1.11 mM) into 10 mM L-malate solution at 25 °C are shown at the top; integrated and dilution corrected peaks are fit to a dimer dissociation model and are shown at the bottom. The data underlying this Figure can be found in S1 Data.

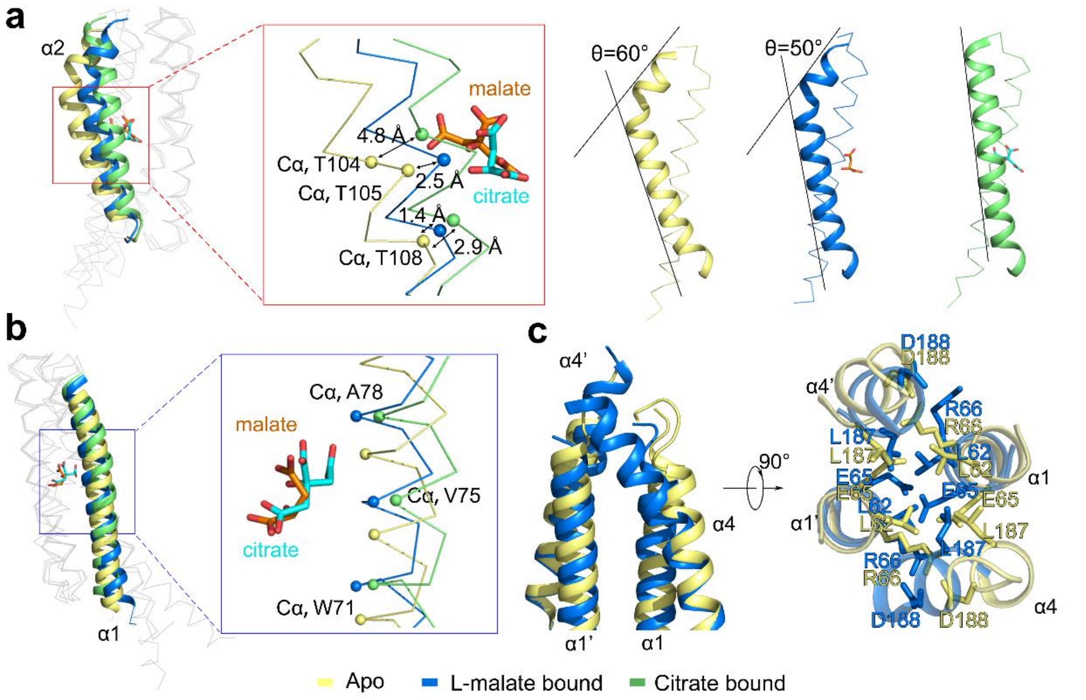

Fig D. Structural comparison of apo-, citrate-bound and malate-bound MCP2201-LBD. (a) Movements of residues T104, T105, and T108 of helix α2 towards the ligand and the bend angles of the C-terminus of helix α2 in apo- (yellow, PDB accession code 5XUA), malate-bound (blue, PDB accession code 7WRM) and citrate-bound (green, PDB accession code 6ITS) structures. (b) Displacements of helix α1 away from the membrane upon ligand binding. (c) Packing of the N-terminus of helix α1 and the C-terminus of helix α4 in the dimeric interface.

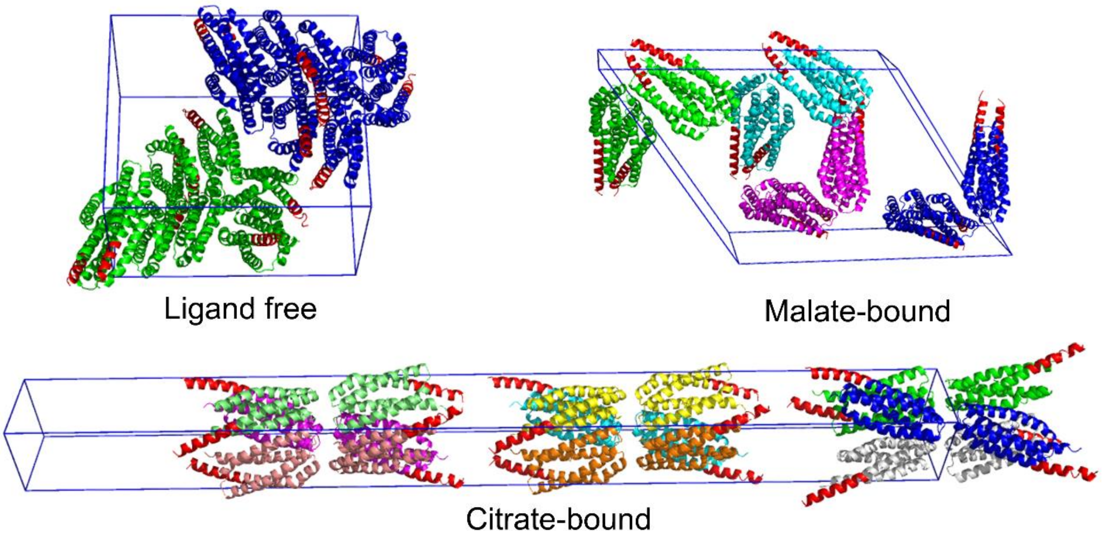

Fig E. The crystal packing of the three LBD states. Ligand-free (PDB entry 5XUA), malate-bound (PDB accession code 7WRM) and citrate-bound MCP2201LBD (PDB accession code 6ITS) were shown. Blue box represents the crystal cell, each color represents the asymmetric unit, and the red color represents the C-terminus of helix α4.

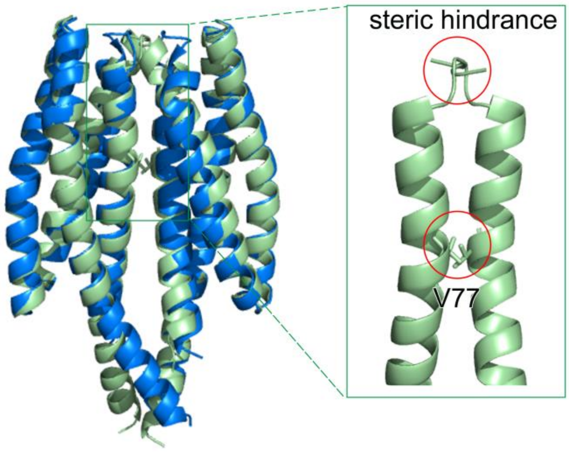

Fig F. Citrate binding induces steric hindrance preventing MCP2201 dimerization. Two citrate-bound MCP2201LBD monomers (green, PDB accession code 6ITS) are superposed with a L-Malate-bound dimer (blue, PDB accession code 7WRM). Two steric hindrances are identified by red circles: Val77 and the loop between helix α1 and α2.

Table A. Strains and plasmids used in this study.

Strains/plasmids	Relevant genotype or description	Source
**Strains**		
Comamonas testosteroni		
CNB-1		[1]
CNB-1Δ20	All putative chemoreceptor genes were disrupted in strain CNB-1	[2]
Escherichia coli		
DH5α	F^- *φ*80d *lacZ*ΔM15 Δ (*lacZYA-argF*) U169 *rec*A1 *end*A1 *hsd*R17(r_K^- m_K^+) *sup*E44 λ- *thi*-1 *gyr*A96 *rel*A1 *pho*A; host for DNA manipulations	[3]
BL21(DE3)	F^- *omp*T *hsd*S_B(r_B^- m_B^-) *gal dcm* (DE3)	Novagen
P. aeruginosa		
PAO1	Wild-type strain	[4]
ΔWspA	*wspA* in-frame deletion	[4]
**Plasmids**		
pBBR1MCS-2	Km^r, *lacPOZ*' broad host vector with R type conjugative origin	[5]
pBBR1MCS2-MCP2201-E65AR66A	Carries MCP2201 with E65AR66A mutation	This work
pBBR1MCS2-MCP2201-S69A	Carries MCP2201 with S69A mutation	This work
pBBR1MCS2-MCP2201-N72A	Carries MCP2201 with N72A mutation	This work
pBBR1MCS2-MCP2201-S73A	Carries MCP2201 with S73A mutation	This work
pBBR1MCS2-MCP2201-S73F	Carries MCP2201 with S73F mutation	This work
pBBR1MCS2-MCP2201-S73R	Carries MCP2201 with S73R mutation	This work
pBBR1MCS2-MCP2201-V77W	Carries MCP2201 with V77W mutation	This work
pBBR1MCS2-MCP2201-A80F	Carries MCP2201 with A80F mutation	This work
pBBR1MCS2-MCP2201-A80W	Carries MCP2201 with A80W mutation	This work
pBBR1MCS2-MCP2201-A84F	Carries MCP2201 with A84F mutation	This work
pBBR1MCS2-MCP2201-A84W	Carries MCP2201 with A84W mutation	This work
pBBR1MCS2-MCP2201-Tar	Carries MCP2201 spanning residues 1-206 and Tar spanning residues 196-553	This work
pHERD20T		
pHERD20T- MCP2201-WspA	Carries MCP2201 spanning residues 1-291 and WspA spanning residues 280-542	This work
pET22b		
pET22b-*mcp2201*LBD	pET22b derivative for expression of MCP2201LBD, spanning residues 58-203 and used for L-malate-bound form structure determination.	This work

Table B. Data collection and refinement statistics for MCP2201LBD structure.

	MCP2201LBD (L-malate-bound)
**Data collection**	
Space group	P 2_1 2_1 2_1
Cell dimensions	
a, b, c (Å)	47.42, 59.84, 98.06
α, β, γ (°)	90.00, 90.00, 90.00
Resolution (Å)	42.70–1.80 (1.90–1.80)
R_{merge}	0.101 (0.698)
I/σ(I)	16.5 (3.5)
$CC_{1/2}$	99.8 (88.5)
Completeness (%)	98.8 (97.6)
Redundancy	11.8 (10.7)
**Refinement**	
No. reflections	26185
R_{work}/R_{free}	0.1739 / 0.2053
No. Non-H atoms	
Protein	2187
Ligand/ion	26
Water	217
B factors	
Protein	26.1
Ligand/ion	22.5
Water	37.7
R.m.s. deviations	
Bond lengths (Å)	0.006
Bond angles (°)	0.804
Ramachandran plot	
Favored	97.8%
Allowed	2.2%
Outliers	0.0%

Legend for Data S1

The numerical values underlying those figures in main text and supplementary information are deposited in S1_Data.xlsx file. The data include:

1) Chemotactic responses of CNB-1Δ20/MCP2201 and CNB-1Δ20 in Figure 1b;
2) Chemotactic responses of CNB-1Δ20/MCP2201 to citrate and malate using the transwell chemotaxis assay in Figure 1c;
3) Chemotaxis of E. coli strain harboring MCP2201-Tar (2201-Tar) or not (WT E. coli) to malate on the gradient soft-agar plate in Figure 1e;
4) Biofilm formation of *P. aeruginosa* strains harboring MCP2201-WspA (2201-WspA) or not (PA) in Figure 1f;
5) Chemotactic responses to malate on the gradient soft-agar plates by CNB-1Δ20 cells harboring MCP2201 mutants in Figure 2c;
6) Analytical ultracentrifugation assays of ligand-free, malate-bound (10 mM), and citrate-bound (10 mM) MCP2201-LBD in Figure 3a;
7) ITC assays of MCP2201-LBD oligomer dissociation in the absence and in the presence of citrate and malate in Figure 3b;
8) Chemotactic responses to malate on the gradient soft-agar plate by CNB-1Δ20 cells harboring MCP2201 mutants in Figure 3e;
9) ITC assays of MCP2201-LBD mutants' malate affinity in Figure B;
10) ITC assays of MCP2201-LBD mutants' oligomer dissociation in the presence of malate in Figure C;
11) Chemotactic responses to citrate on the gradient soft-agar plate by CNB-1Δ20 cells harboring MCP2201 mutants in Figure A.

References

1. Wu JF, Jiang CY, Wang BJ, Ma YF, Liu ZP, Liu SJ. Novel partial reductive pathway for 4-chloronitrobenzene and nitrobenzene degradation in *Comamonas* sp. strain CNB-1. Appl Environ Microbiol. 2006;72(3):1759-65.
2. Ni B, Huang Z, Fan Z, Jiang CY, Liu SJ. *Comamonas testosteroni* uses a chemoreceptor for tricarboxylic acid cycle intermediates to trigger chemotactic responses towards aromatic compounds. Mol Microbiol. 2013;90(4):813-23.
3. Hanahan D. Studies on transformation of *Escherichia coli* with plasmids. J Mol Biol. 1983;166(4):557-80.
4. Xu A, Wang D, Wang Y, Zhang L, Xie Z, Cui Y, et al. Mutations in surface-sensing receptor WspA lock the Wsp signal transduction system into a constitutively active state. Environ Microbiol. 2021 https://doi.org/10.1111/462-2920.15763.
5. Kovach ME, Elzer PH, Hill DS, Robertson GT, Farris MA, Roop RM, 2nd, et al. Four new derivatives of the broad-host-range cloning vector pBBR1MCS, carrying different antibiotic-resistance cassettes. Gene. 1995;166(1):175-6.
